# Parametric analysis of the transmission dynamics during indigenous aggregated outbreaks caused by five SARS-CoV-2 strains in Nanjing, China

**DOI:** 10.3389/fpubh.2024.1358577

**Published:** 2024-03-08

**Authors:** Tao Ma, Cong Chen, Junjun Wang, Hengxue Wang, Yueyuan Zhao, Yuanzhao Zhu, Zikang Yan, Songning Ding, Jie Ding

**Affiliations:** ^1^Department of Acute Infectious Diseases Control and Prevention, Nanjing Municipal Center for Disease Control and Prevention, Nanjing, China; ^2^Wujin District Center for Disease Control and Prevention, Changzhou, China; ^3^Jiangsu Field Epidemiology Training Program, Jiangsu Provincial Centre for Disease Control and Prevention, Nanjing, China

**Keywords:** COVID-19, SARS-CoV-2, transmission dynamics, parametric analysis, indigenous aggregated outbreaks

## Abstract

**Background:**

SARS-CoV-2 strains have been of great concern due to their high infectivity and antibody evasion.

**Methods:**

In this study, data were collected on indigenous aggregated outbreaks in Nanjing from January 2020 to December 2022, caused by five strains including the original strain, the Delta variant, and the Omicron variant (BA.2, BA.5.2, and BF.7). The basic epidemiological characteristics of infected individuals were described and then parametric analysis of transmission dynamics was performed, including the calculation of incubation period, serial interval (SI), the basic reproductive number (R_0_), and the household secondary attack rate (HSAR). Finally, we compared the trends of transmission dynamic parameters of different strains.

**Results:**

The incubation period for the original strain, the Delta variant, Omicron BA.2, Omicron BA.5.2, and Omicron BF.7 were 6 d (95% CI: 3.5–7.5 d), 5 d (95% CI: 4.0–6.0 d), 3 d (95% CI: 3.0–4.0 d), 3 d (95% CI: 3.0–3.0 d), and 2 d (95% CI: 2.0–3.0 d), respectively; Also, the SI of the five strains were 5.69 d, 4.79 d, 2.7 d, 2.12 d, and 2.43 d, respectively. Notably, the incubation period and SI of the five had both a progressive shortening trend (*p* < 0.001); Moreover, R_0_ of the five were 2.39 (95% CI: 1.30–4.29), 3.73 (95% CI: 2.66–5.15), 5.28 (95% CI: 3.52–8.10), 5.54 (95% CI: 2.69–11.17), 7.39 (95% CI: 2.97–18.76), with an increasing trend gradually (*p* < 0.01); HSAR of the five were 25.5% (95% CI: 20.1–31.7%), 27.4% (95% CI: 22.0–33.4%), 42.9% (95% CI: 34.3–51.8%), 53.1% (95% CI: 45.0–60.9%), 41.4% (95% CI, 25.5–59.3%), also with an increasing trend (*p* < 0.001).

**Conclusion:**

Compared to the original strain, the incubation period and SI decreased while R_0_ and HSAR increased, suggesting that transmission in the population was faster and the scope of the population was wider. Overall, it’s crucial to keep implementing comprehensive measures like monitoring and alert systems, herd immunization plans, and outbreak control.

## 1 Introduction

Severe Acute Respiratory Syndrome Coronavirus 2 (SARS-CoV-2), first identified in December 2019, is constantly changing throughout the global pandemic of coronavirus disease 2019 (COVID-19) (1). SARS-CoV-2 has evolved from the original strain of the first epidemic to the Beta, Delta, and now the predominantly endemic Omicron strains, which are of great concern due to the increasing transmission and immune escape ability (2).

Several localized outbreaks in clusters occurred in Nanjing between 2020 and 2022, with different pathogenic strains, including the original strain, the Delta variant (B.1.617.2) and the Omicron variant (BA.2, BA.5.2, and BF.7) (3). Therefore, it is of great public health significance to optimize the prevention and control strategy, the combination of government control, and personal protection measures according to the transmission characteristics of the epidemic in Nanjing.

Transmission dynamics parameters can reflect the speed, scale, and prevention and control effects of pathogen transmission in the population, and are often used to study the transmission trends and transmission risks of COVID-19 epidemics around the world during epidemics (4–6).

In this study, we collected case information through epidemiological surveys to understand the epidemiological characteristics of the outbreaks and analyzed the transmission dynamics parameters of different strains. The aim of this study is to provide a scientific basis for the development of prevention and control strategies in the context of continuous virus mutation.

## 2 Methods

### 2.1 Data collection

We retrospectively collected data on all confirmed cases of six localized outbreaks caused by five strains of original strain, Delta variant, and Omicron variant (BA.2, BA.5.2, and BF.7) during the period of the “Dynamic Zeroing” strategy in Nanjing, Jiangsu Province, from January 2020 to December 2022, including case information, age, sex, clinical typing, vaccination status, laboratory test results, initial screening positive cycle threshold (Ct) value, onset date, initial exposure date, last exposure date, and initial screening positive date. The data in this study were from epidemiological investigations, which were updated and managed in the National Infectious Diseases Information Reporting Management System and stored in Nanjing Center for Disease Control and Prevention.

### 2.2 Case definition

Cases were defined according to the “Protocol for Prevention and Control of COVID-19” released by the National Health Commission of the People’s Republic of China (7), and SARS-CoV-2-infected cases were those with positive nucleic acid results using reverse transcription-polymerase chain reaction (RT-PCR) targeting ORF1ab and N genes of SARS-CoV-2. Samples were collected including nasal and oropharyngeal swabs, sputum, and other respiratory tract specimens.

### 2.3 Diagnostic criteria

The diagnostic criteria to determine COVID-19 severity in this study were based on the diagnosis and treatment protocol for COVID-19 issued by the National Health Commission (8, 9). Clinical classification is as follows: asymptomatic infections, moderate cases, severe cases and critical case. Confirmed cases are classified according to the severity of infection into moderate cases, severe cases and critical cases. Asymptomatic infections are characterized as individuals who test positive for the novel coronavirus through pathogen examination but do not display associated clinical symptoms or signs.

### 2.4 Calculation of transmission dynamics parameters

According to the case information, 36 (original strain), 72 (delta), 76 (omicron BA.2), 124 (omicron BA.5.2), and 103 (omicron BF.7) “transmission pairs” were established for the five strains of infector-infected, respectively.

The incubation period refers to the time interval between the pathogen’s invasion of an individual and the onset of symptoms (10). The incubation period was calculated using infected individuals with a single exposure in this study.

Serial interval (SI), the time between symptoms in a case and their infector (11), was calculated by R software using the fitted pairs of transmitters with a clear intergenerational transmission relationship. Of these, the original strain, Omicron BA.2 and Omicron BF.7 followed a gamma distribution, the Delta strain followed a Weibull distribution and the Omicron BA.5.2 strain followed a lognormal distribution.

Basic reproductive number (R_0_) measures the average number of secondary cases in a susceptible population infected by a typical primary case during the infection period (12). Referring to previous literature, this study applied an exponential growth (EG) model to calculate R_0_ by using the incidence curves and serial interval of infected individuals before the outbreaks were detected (3).

In addition, household secondary attack rates (HSAR), which is the percentage of secondary cases that are following primary cases among their household contacts. In this study, we collected the number of household secondary infected persons and the number of household contacts to calculate the household secondary attack rates.

### 2.5 Statistical analysis

Data were recorded using Excel software. Data were analyzed using R 4.1.3 software to calculate parameters including incubation period, SI, R_0_, and HSAR. Simple linear regression (continuous variable) and chi-squared test of trend (categorical variable) were utilized to compare the trends of transmission dynamic parameters of different strains with the time order of variation. The statistical significance level was *α* = 0.05.

## 3 Results

### 3.1 Characteristics of the epidemic of COVID-19

**Figure 1:**
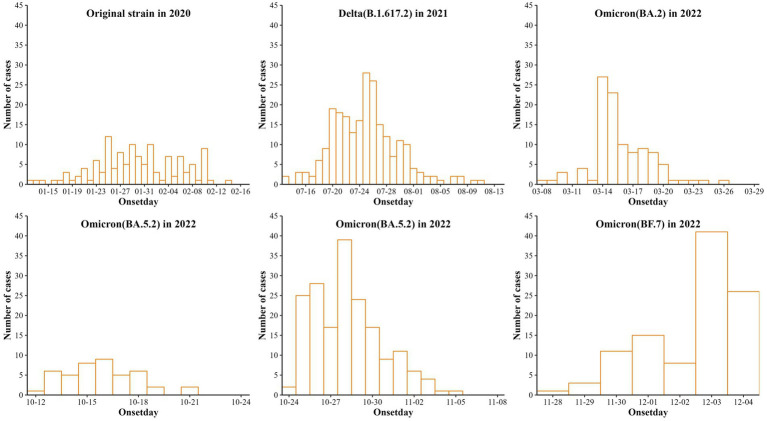
Reporting of six cases of aggregated outbreaks of COVID-19 caused by 5 strains in Nanjing.

As shown in Figure 1, the six aggregated outbreaks in Nanjing reported 126 infected cases (original strain), 235 cases (Delta), 105 cases (Omicron BA.2), 44 cases (Omicron BA.5.2), 184 cases (Omicron BA.5.2) and 105 cases (Omicron BF.7). The first to last case onset dates are January 12–February 17, 2020; July 13–August 12, 2021; March 8–March 26, 2022; October 12–October 21, 2022; October 24–November 5, 2022; and November 28, 2022 (policy adjustment).

Details of reported infections by strains are shown in Table 1. It also shows that the number and percentage of each sex were 65 (51.6), 94 (40.0), 44 (41.9), 32 (72.7), 127 (69.0), 50 (47.6) for males and 61 (48.4), 141 (60.0), 61 (58.1), 12 (27.3), 57 (31.0), 55 (52.4) for females. Ages [Median (IQR)] were 44 (30–58), 44 (34–53), 37 (28–56), 33 (20–46), 46 (32–57), and 60 (48–66). The number and percentage of unvaccinated persons were 126 (100.0), 55 (23.4), 11 (doi: 10.5), 3 (6.8), 8 (4.4), 14 (13.3). First positive nucleic acid Ct values were 30.2 (25.0–33.3), 24.5 (19.8–29.2), 28.9 (21.9–33.4), 32.4 (28.3–35.1), 31.1 (26.2–34.4), and 29.8 (25.3–33.2) for the N gene [Median (IQR)] and 29.8 (24.8–32.6), 25.7 (21.4–30.7), 29.1 (23.5–34.5), 33.2 (29.8–36.1), 32.1 (27.6–35.7), 30.4 (26.4–33.7) for Orf1ab gene [Median (IQR)].

**Table 1:** Epidemiological characteristics of outbreaks in Nanjing.

	Original strain	Delta B.1.617.2	Omicron BA.2	Omicron BA.5.2	Omicron BA.5.2	Omicron BF.7
Age group (years)						
<18	11 (8.7)	27 (11.5)	17 (16.2)	5 (11.4)	17 (9.2)	0 (0)
18–39	46 (36.5)	55 (23.4)	40 (38.1)	23 (52.3)	57 (31.0)	19 (17.9)
40–60	39 (31.0)	120 (51.1)	25 (23.8)	14 (31.8)	75 (40.8)	33 (31.1)
>60	30 (23.8)	33 (14.0)	23 (21.9)	2 (4.5)	35 (19.0)	53 (50.9)
Age[Median (IQR)]	44 (30–58)	44 (34–53)	37 (28–56)	33 (20–46)	46 (32–57)	60 (48–66)
Gender						
Male (%)	65 (51.6)	94 (40.0)	44 (41.9)	32 (72.7)	127 (69.0)	50 (47.6)
Female (%)	61 (48.4)	141 (60.0)	61 (58.1)	12 (27.3)	57 (31.0)	55 (52.4)
Case type						
Reported	126	235	105	44	184	105
Asymptomatic	33 (26.2)	0 (0)	100 (95.2)	12 (27.3)	71 (38.6)	11 (12.4)
Confirmed	93 (73.8)	235 (100.0)	5 (4.8)	32 (72.7)	113 (61.4)	78 (87.6)
Moderate	83	225	5	32	113	78
Severe/Critical	10	10	0	0	0	0
Vaccination dose						
0	126 (100.0)	55 (23.4)	11 (doi:10.5)	3 (6.8)	8 (4.4)	14 (13.3)
1	0 (0)	52 (22.1)	4 (3.8)	0 (0)	4 (2.2)	2 (1.9)
2	0 (0)	127 (54.1)	46 (43.8)	13 (29.5)	56 (30.4)	20 (19.1)
3	0 (0)	1 (0.4)	44 (41.9)	28 (63.7)	116 (63.0)	69 (65.7)
Ct value						
N[Median (IQR)]	30.2 (25.0–33.3)	24.5 (19.8–29.2)	28.9 (21.9–33.4)	32.4 (28.3–35.1)	31.1 (26.2–34.4)	29.8 (25.3–33.2)
O[Median (IQR)]	29.8 (24.8–32.6)	25.7 (21.4–30.7)	29.1 (23.5–34.5)	33.2 (29.8–36.1)	32.1 (27.6–35.7)	30.4 (26.4–33.7)

### 3.2 Transmission dynamics parameter fitting for incubation period and serial interval (SI)

**Figure 2:**
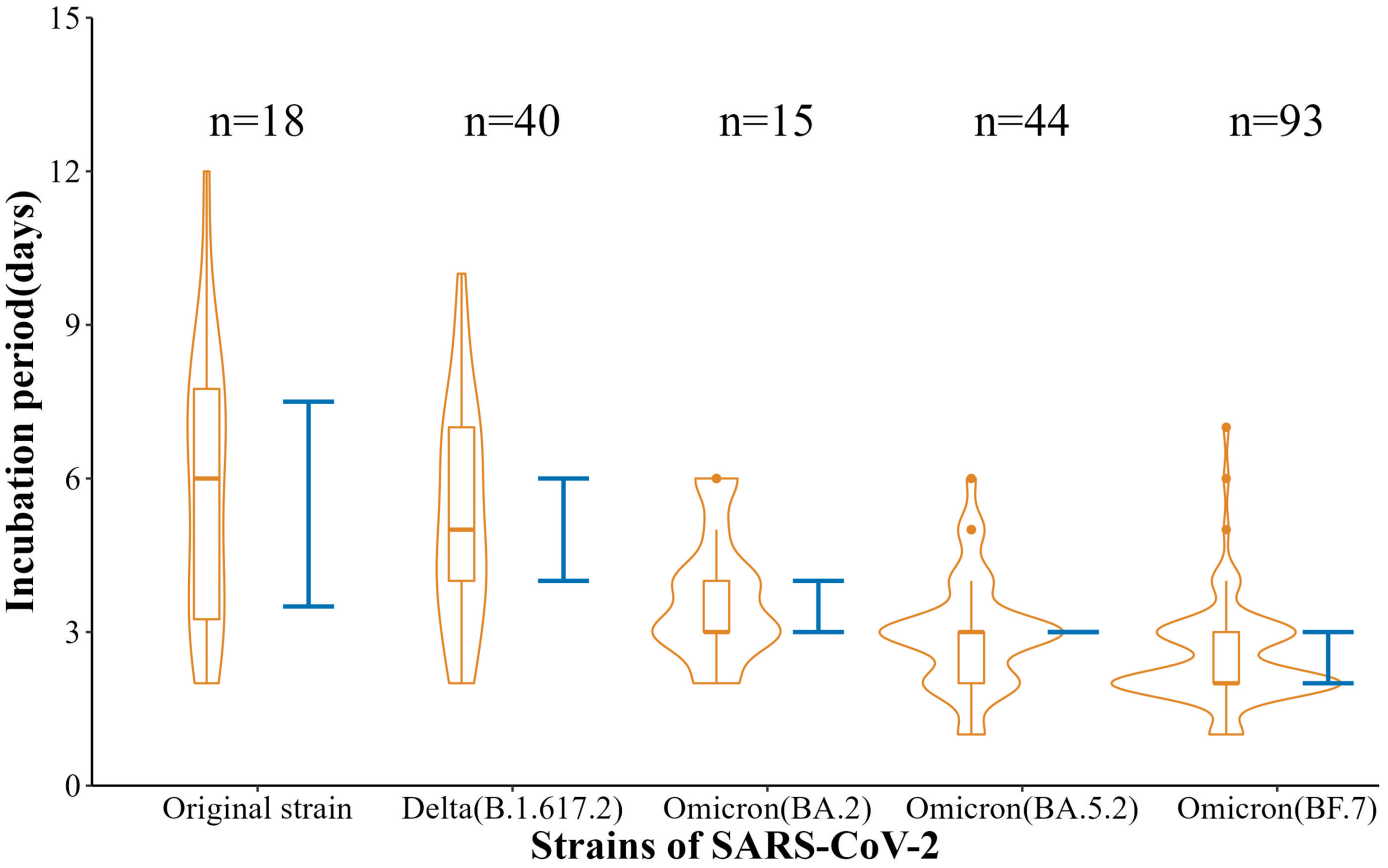
Estimation of incubation period of five SARS-CoV-2 strains in Nanjing.

**Figure 3:**
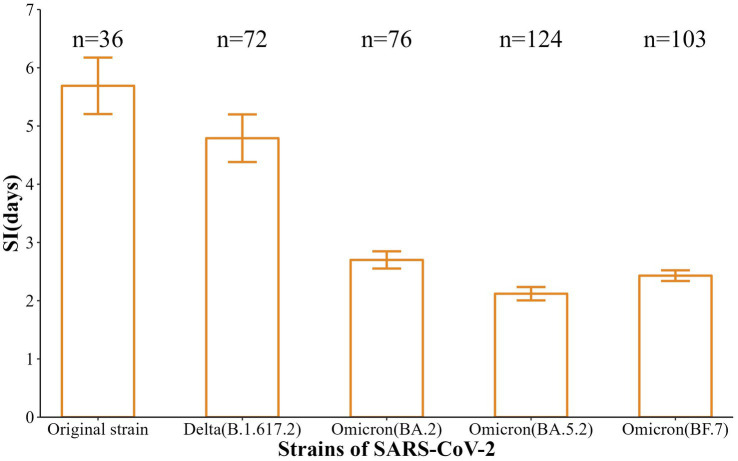
SI estimation results for five SARS-CoV-2 strains in Nanjing.

Also, the incubation periods [Median (Min-Max)] of the five strains were 6 d (2-12 d), 5 d (2-10 d), 3 d (2-6 d), 3 d (1-6 d), and 2 d (1-7 d), with 95% CI of 3.5–7.5 d, 4.0–6.0 d, 3.0–4.0 d, 3.0–3.0 d, and 2.0–3.0 d. These reflected a gradual shortening trend (*t* = −11.88, *p* < 0.001) (Figure 2). In addition, the SI [Mean (SD)] were 5.69 d (2.91 d), 4.79 d (3.47 d), 2.70 (1.29 d), 2.12 d (1.28 d), and 2.43 d (0.93 d), respectively, with a gradual shortening trend (*t* = −doi: 10.63, *p* < 0.001) (Figure 3).

### 3.3 Calculation of basic reproductive number (R_0_) and household secondary attack rates (HSAR)

**Figure 4:**
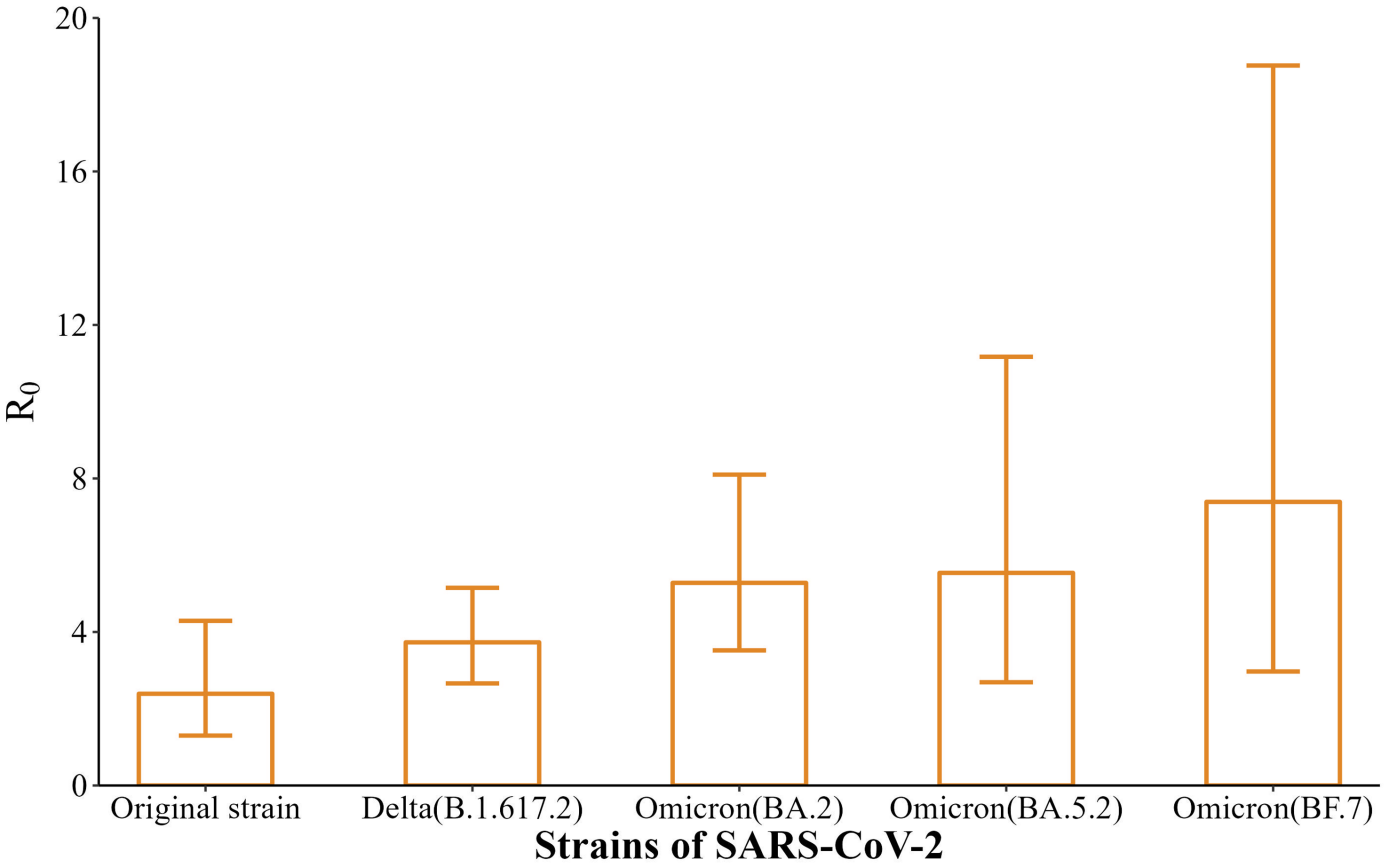
Calculation results of R_0_ for five SARS-CoV-2 strains in Nanjing.

In this study, the EG model was applied, using the incidence curves and SI of infected individuals before the outbreak was detected (before intervention) to calculate R_0_. As shown in Figure 4, the R_0_ of the five strains were 2.39 (95% CI: 1.30–4.29), 3.73 (95% CI: 2.66–5.15), 5.28 (95% CI: 3.52–8.10), 5.54 (95% CI: 2.69–11.17), and 7.39 (95% CI: 2.97–18.76), with a gradually increasing trend (*t* = 9.44, *p* < 0.01).

In addition, the results showed that the HSAR of the five strains were 25.5% (95% CI: 20.1–31.7%), 27.4% (95% CI: 22.0–33.4%), 42.9% (95% CI: 34.3–51.8%), 53.1% (95% CI: 45.0–60.9%), and 41.4% (95% CI: 25.5–59.3%), which were in an increasing trend (*χ*2trend = 32.28, *p* < 0.001), as detailed in Table 2.

**Table 2:** Number of household contacts, number of household secondary infected persons, and household secondary attack rates for the five SARS-CoV-2strains.

SARS-CoV-2 strain	Original strain	Delta B.1.617.2	BA.2	BA.5.2	BF.7
Number of household contacts	216	234	119	147	29
Number of household secondary infected persons	55	64	51	78	12
Household secondary attack rates [% (95% CI)]	25.5 (20.1–31.7)	27.4 (22.0–33.4)	42.9 (34.3–51.8)	53.1 (45.0–60.9)	41.4 (25.5–59.3)

## 4 Conclusion

SARS-CoV-2 have mutated significantly since their first discovery. Notably, Omicron variant strain is the most highly mutated variant and has become the most predominant epidemic strain worldwide (13, 14). Therefore, in this study, we comparatively analyzed the epidemiological characteristics and transmission dynamics parameters of local aggregated outbreaks caused by five SARS-CoV-2 variant strains in Nanjing, aiming to provide a reference for recognizing the transmission capacity of SARS-CoV-2 strains (especially the Omicron variant).

The original strain is the most common strain in 2021 in Nanjing, the most common strain is the Delta strain in 2022, and it is dominated by the different variants of Omicron in 2023. Omicron BA.5.2 and BF.7 are the main reasons for the surge in cases after mid-November 2022. In this study, the median incubation periods of five SARS-CoV-2 strains in Nanjing, i.e., the original strain, Delta, Omicron BA.2, Omicron BA.5.2, and Omicron BF.7, were found to be 6, 5, 3, 3, and 2 days. There were some differences in the incubation periods between these five types of variant infection, and the incubation period of variant BF.7 infection was much shorter than others, which is consistent with recent research found in Beijing or Shenzhen (15–17). In short, participants with Omicron had a shorter incubation period than those with the historical strains, which has been recognized in many studies (18, 19), suggesting the need for rapid response and strict disease control in the event of an epidemic, especially for Omicron.

Normally, the faster the infected person is identified and isolated, the shorter the SI is and the lower the chance of virus transmission (20). The results showed that the median SI of the five strains that caused the six aggregated outbreaks in Nanjing were 5.69, 4.79, 2.70, 2.12, and 2.43 days, respectively, which showed a trend of gradual shortening, and all of them were lower than that of the initial outbreak in Wuhan (7.5 days) (21). In conclusion, the mutation of the SARS-CoV-2 strains, the incubation period showed a trend of gradual shortening, and the speed of transmission became faster.

R_0_ is used as an important indicator of the transmission ability of SARS-CoV-2 (22), and the results of EG model fitting in this study showed that the R_0_ of the five strains were 2.39, 3.73, 5.28, 5.54, and 7.39, respectively. The results suggest that the evolved strains (Delta, Omicron BA.2, Omicron BA.5.2, Omicron BF.7) were more infectious than the original strain in Nanjing, resulting in rapid transmission of COVID-19 among susceptible populations. Accumulating studies have shown that R_0_ tends to decline with the development of the COVID-19 epidemic and the implementation of interventions (23, 24). This suggests that in the later stage of prevention and control, the gradually increasing R_0_ of the virulent strains should be taken into full consideration and appropriate interventions should be selected to control the spread of the epidemic.

In addition, family aggregation is important in epidemic prevention, control and disposal (25). The number of household contacts and the number of household secondary infected persons were collected in order to calculate the HSAR, and the HSAR of the five strains were 25.5, 27.4, 42.9, 53.1, and 41.4%, respectively, which showed an increasing trend. The estimated HSAR of the five SARS-CoV-2 variant strains in Nanjing exceed by far in Wuhan (the HASR of 15.6% between Dec 2019 and April 2020), Guangzhou (17.7% between Jan and Feb, 2020), and Beijing (23% from Feb to Mar, 2020) found by previous studies (26–28), but was similar to that in Zhejiang province (31.6% from Jan to Feb, 2020) (29). Also, HSAR estimates in mainland China have tended to be higher than those for other locations like doi: 10.5% in the USA (between Jan and Feb, 2020) and 4.6% in Taiwan (from Jan to Apr, 2020) (30, 31). The HSAR across different regions is probably due to differences in control measures, surveillance practices, and crowdedness in households. Overall, there is no doubt that persons living together in a family with longer contact time, frequent contact opportunities and closer contacts had the highest incidence rate in family aggregation of outbreaks. Therefore, with the gradual increase of HSAR, it suggests that monitoring and control should be implemented as early as possible for family members living together in order to effectively contain the spread of the epidemic.

This study has some limitations. The study only analyzed data in Nanjing until November 2022 due to the termination of large-scale nucleic acid testing. The results may have some degree of bias. Our data better represent the incidence of Nanjing, but more surveillance data are needed for the future spread and pathogenic impact.

In summary, from the original strain, Delta, to Omicron, the incubation period and SI were progressively shortened while R_0_ and HSAR were progressively increased, suggesting a faster spread and reaching a wider range of people in a population. Under the “Dynamic Zeroing” strategy, the rapid spread of the epidemic was effectively interrupted. The mutation of the virus has increased the pressure to prevent and control the epidemic, and comprehensive strategies such as surveillance and early warning, mass immunization, and outbreak control should continue to be implemented.

## Data availability statement

The raw data supporting the conclusions of this article will be made available by the authors, without undue reservation.

## Ethics statement

The epidemiological investigation was undertaken according to the Protocol for Prevention and Control of COVID-19. Individual-identifying information was not retained in analytic data sets. The studies were conducted in accordance with the local legislation and institutional requirements. Written informed consent for participation was not required from the participants or the participants’ legal guardians/next of kin in accordance with the national legislation and institutional requirements.

## Author contributions

TM: Conceptualization, Formal analysis, Supervision, Writing – original draft, Writing – review & editing. CC: Formal analysis, Writing – original draft, Writing – review & editing. JW: Data curation, Formal analysis, Investigation, Writing – review & editing. HW: Data curation, Formal analysis, Investigation, Writing – review & editing. YZha: Data curation, Investigation, Writing – review & editing. YZhu: Data curation, Investigation, Writing – review & editing. ZY: Data curation, Investigation, Writing – review & editing. SD: Conceptualization, Project administration, Supervision, Writing – review & editing. JD: Conceptualization, Project administration, Supervision, Writing – review & editing.
